# A cost-minimization analysis of diuretic-based antihypertensive therapy reducing cardiovascular events in older adults with isolated systolic hypertension

**DOI:** 10.1186/1478-7547-3-2

**Published:** 2005-01-25

**Authors:** G John Chen, Luigi Ferrucci, William P Moran, Marco Pahor

**Affiliations:** 1 Department of Public Health Sciences, Wake Forest University School of Medicine, Winston-Salem, NC, USA; 2 Laboratory of Clinical Epidemiology, INRCA Geriatric Department, Florence, Italy; 3 Department of Internal Medicine, Wake Forest University School of Medicine, Winston-Salem NC, USA

## Abstract

**Background:**

Hypertension is among the most common chronic condition in middle-aged and older adults. Approximately 50 million Americans are currently diagnosed with this condition, and more than $18.7 billion is spent on hypertension management, including $3.8 billion for medications. There are numerous pharmacological agents that can be chosen to treat hypertension by physicians in clinical practices. The purpose of this study was to assess the cost of alternative antihypertensive treatments in older adults with isolated systolic hypertension (ISH).

**Method:**

Using the Systolic Hypertension in the Elderly Program (SHEP) and other data, a cost-minimization analysis was performed. The cost was presented as the cost of number-needed-to treat (NNT) of patients for 5 years to prevent one adverse event associated with cardiovascular disease (CVD).

**Result:**

It was found that the cost of 5 year NNT to prevent one adverse CVD event ranged widely from $6,843 to $37,408 in older patients with ISH. The incremental cost of the 5 year NNT was lower to treat older patients in the very high CVD risk group relative to patients in the lower CVD risk group, ranging from $456 to $15,511. Compared to the cost of the 5 year NNT of other commonly prescribed antihypertensive drugs, the cost of SHEP-based therapy is the lowest. The incremental costs of the 5 year NNT would be higher if other agents were used, ranging from $6,372 to $38,667 to prevent one CVD event relative to SHEP-based drug therapy.

**Conclusion:**

Antihypertensive therapy that is diuretic-based and that includes either low-dose reserpine or atenolol is an effective and relatively inexpensive strategy to prevent cardiovascular events in older adults with isolated systolic hypertension. Use of the diuretic-based therapy is the most cost-effective in patients at high risk for developing cardiovascular disease.

## Background

Hypertension is among the most common chronic conditions in middle-aged and older adults. Approximately 50 million Americans are currently diagnosed with this condition, and more than $18.7 billion is spent on hypertension management, including $3.8 billion for medications[1].

Treatment of hypertension can significantly decrease the risk of developing CVD [2,3]. The SHEP and other studies have demonstrated the great potential of antihypertensive treatments to significantly reduce the number of cardiovascular events in elderly patients [4-10]. This, in turn, may reduce the costs associated with this chronic condition. Based on the SHEP study, it is estimated that 24,000 strokes, 44,000 major cardiovascular events, and 84,000 admissions to the hospital could be prevented over a 5-year period [7].

Currently, primary care physicians can choose from numerous pharmacological agents to treat hypertension. The commonly used antihypertensive drug classes include diuretics, beta-blockers, angiotensin-converting enzyme (ACE) inhibitors, alpha-blockers, and calcium channel blockers. Selection of an evidence-based therapy with demonstrated efficacy, safety, and low cost has important economic implications. The purpose of this study was to: 1) assess cost of the SHEP-based antihypertensive treatment to prevent adverse events associated with CVD, including death, stroke, myocardial infarction, and heart failure; and 2) to compare cost of the SHEP-based treatment to the costs of other commonly used antihypertensive agent treatments.

## Method

The SHEP trial is a randomized, double-blind, placebo-controlled clinical trial sponsored by the National Heart, Lung, and Blood Institute and the National Institute on Aging that tested the efficacy of diuretic-based stepped-care antihypertensive drug treatment of isolated systolic hypertension (ISH) to prevent strokes [4].

### Study Population

The study subjects consisted of community-dwelling men and women 60 years and older who had isolated systolic hypertension, defined as an average systolic blood pressure (SBP) ≥ 160 mm Hg and an average diastolic blood pressure (DBP) < 90 mm Hg over 2 baseline visits. The primary endpoint of the trial was combined nonfatal and fatal stroke over a 5-year period. Secondary endpoints included nonfatal myocardial infarction (MI) plus fatal coronary heart disease (CHD) and major cardiovascular disease (CVD) morbidity and mortality. A total of 2,365 and 2,371 persons were randomized into the treatment and placebo group of the study respectively.

Subjects who met the preliminary blood pressure (BP) eligibility criteria at the initial contact visit were referred to SHEP clinics for the baseline visits. At the baseline visits, subject's demographics, medical conditions, health behaviors, and cardiovascular risk factors were obtained. Methods of these measurements have been reported^4^. Fasting blood samples were analyzed at a central laboratory, including serum glucose, lipid levels, creatinine, uric acid, sodium, and potassium.

Of the 4,736 SHEP participants, 4,189 were included in this analysis. The 547 participants were excluded either because of missing data concerning CVD risk factors (n = 283) or with previous CHD or stroke (n = 264). These 547 excluded subjects had similar age, sex, race, and other characteristics as those who were included in this analysis.

### Intervention

A stepped-care treatment approach was used, with the goal for individuals with SBP >180 mm Hg to reduce to **<**160 mm Hg and for those with SBP between 160 and 179 mm Hg to have a reduction of at least 20 mm Hg. All participants were given chlorthalidone, 12.5 mg/d, or matching placebo (step 1 and dose 1 medication). Drug dosage (step 1 and dose 2 medication) was doubled, 25 mg/d, for participants failing to achieve the SBP goal at the follow-up visits. If the SBP goal was not reached at the maximal dose of step 1 medication, atenolol, 25 mg/d, or matching placebo was added (step 2 and dose 1 medication). When atenolol was contraindicated, reserpine, 0.05 mg/d, or matching placebo could be substituted. When required to reach the blood pressure goal, the dosage of the step 2 drug could be doubled (atenolol 50 mg/d or reserpine 0.10 mg/d, step 2 and dose 2 medication). Potassium supplements were given to all participants who had serum concentration below 3.5 mm0l/L at two consecutive visits. The SHEP participants were followed up monthly until SBP reached the goal or until the maximum level of stepped-care treatment was reached [4,7]

### Ascertainment of Outcome Events

The present analysis focused on five types of events: 1) death; 2) first-occurring major cardiovascular event, including stroke, MI, or heart failure; 3) first-occurring stroke; 4) first-occurring MI; and 5) first clinical diagnosis of congestive heart failure (CHF). The adjudication and clarification of the events was done by a panel of three physicians blinded to treatment assignment and blood pressure status. Members of the panel reviewed the documentation of new cardiovascular events over the study period and adjudicated outcome events according to predetermined criteria. [4]

### Calculation of Global CVD Risk Scores

Information at the baseline on age, sex, total cholesterol, high density lipid (HDL) cholesterol, systolic blood pressure, diabetes (diabetic vs. non-diabetic), and smoking (current vs. never or past smoking) were used to calculate an *a priori *global score for the risk of developing future cardiovascular events, according to the Multiple Risk Factor Assessment Equation jointly proposed by the American Heart Association and the American College of Cardiology.[11] The equation assigns scores to major risk factors, using cut points that were originally developed using data on incident CHD from the Framingham study. A global CVD risk score ranging from -17 to +22 was obtained by adding the subscores. Higher values reflect a more unfavorable risk profile. Because the equation does not provide the age score for persons ≥ 75 years of age (28.5% of the SHEP study population), one additional point was assigned to men and women in this age group. Based on the global cardiovascular risk score, participants were classified into one of four CVD risk groups: low, medium, high and very high.

### Calculation of Costs

The methods of economic evaluation include cost-effectiveness analysis, cost-utility analysis, and cost-benefit analysis, which can be used to assess the trade-off between costs and benefits in choices of antihypertensive treatment regimens. The primary aim of this analysis was to examine cost of the diuretic-based antihypertensive drug intervention in the SHEP trial. A cost-minimization analysis is a special type of cost-effectiveness analysis. It can be used to compare cost difference among competing alternatives of antihypertensive drug treatments when these treatments are medically equivalent. In this study, we used cost-minimization analyses to compare costs and incremental costs of NNT for 5 years to prevent one adverse event related to CVD among antihypertensive treatment regimens. The perspective of this economic evaluation was that of a national health insurance system.

We used the number-needed-to-treat as an unit of common outcome measure in the analysis. The number-needed-to-treat to prevent one adverse outcome has become a widely used measure of treatment benefits derived from the results of clinical trials. The NNT is the reciprocal of the absolute risk reduction (ARR) which is the difference between the proportions with the adverse event in the treatment and placebo groups. The 95% confidence interval of NNT was calculated based on the regression-based method described by Laupacis et al. [12]

The cost specified in the analysis includes the drug acquisition cost of SHEP treatment from the perspective of a national health insurance system. According to the SHEP treatment protocol, the stepped-care was classified into four types of drug treatments: 1) the Step 1 and Dose 1: chlorthalidone 12.5 mg/d; 2) the Step and Dose 2: chlorthalidone 25 mg/d; 3) the Step 2 and Dose 1: chlorthalidone 25 mg/d plus atenolol 25 mg/d or reserpine 0.05 mg/d; and 4) the Step 2 and Dose 2: chlorthalidone 25 mg/d plus atenolol 50 mg/d or reserpine 0.1 mg/d. Direct drug acquisition costs were calculated based on the minimum average wholesale prices (AWP) within drug manufacturers in the year 2000.[13] All drug costs were based on the AWP per unit dose. The expected cost (EC) of the SHEP drug acquisition per patient in 1 year was calculated as follows:

EC = W_1 _× C_1 _+ W_2 _× C_2 _+ W_3 _× C_3 _+ W_4 _× C_4_

The W1, W2, W3, and W4 represent proportions of the participants using the Step 1 and Dose 1, the Step 2 and Dose 2, the Step 2 and Dose 1, and the Step 2 and Dose 2 medication, respectively. C1, C2, C3, and C4 represent the drug acquisition cost of the Step 1 and Dose 1, the Step 2 and Dose 2, the Step 2 and Dose 1, and the Step 2 and Dose 2 medication, respectively. A Monte Carlo method was performed to estimate the average cost and its standard deviation.

To compare the cost of the SHEP-based therapy to other antihypertensive drugs, it was assumed that all antihypertensive drugs in the comparisons have equal efficacy in terms of the NNT for 5 years to prevent one CVD related event. The NNT was calculated based on the method. [12]

All drug costs were expressed as dose-specific cost per patient in 1-year and/or 5-year. Using the approach, costs were calculated for each representative drug based on equipotent doses in terms of blood pressure reduction. [14] The non-SHEP based drugs, including beta-blockers (Atenolol), ACE inhibitors (Enalapril), and calcium channel blockers (Nifedipine), were selected in the analysis according to antihypertensive drug class. These drugs were considered commonly prescribed antihypertensive medications in clinical practices. [14] All costs were adjusted in 2000 constant U.S. dollars using the Consumer Price Index.

In this analysis, we focused on the drug acquisition cost for antihypertensive management. Therefore, the monitoring cost for antihypertensive treatment was not included. Total treatment cost includes antihypertensive drug cost and monitoring cost. The monitoring of treatment in ambulatory care settings including physician visits and laboratory tests have an estimated cost of $284 per patient per year. [14] Total cost of the NNT for 5 years of each drug therapy was calculated by multiplying the NNT for 5 years with the drug acquisition cost for 5 years per patient. The incremental cost is the cost of NNT for 5 years to prevent one adverse event of one alternative less the cost of the base case. In calculations of the incremental costs of the NNT for 5 years by types of outcome, the cost to prevent one stroke which was used as a base case. In calculations of the incremental costs of the NNT for 5 years by risk levels of CVD, the cost to prevent one adverse event of the very high risk level being used as a base case.

## Result

Table 1 shows the expected acquisition cost of the diuretic-based antihypertensive therapies. The step 1 and dose 1 medication was the most used therapy and followed by the step 1 and dose 2 medication. The annual drug acquisition costs of the step 1 and dose 1, the step 1 and dose 2, the step 2 and dose 1 and the step 2 and dose 2 were $10.24, $20.48, and $222.45 respectively. The expected annual drug acquisition cost per patient of the SHEP treatment without potassium supplements was $83 and with potassium supplements was $91. The 5 year annual drug acquisition cost with potassium supplements per patient was $456.

**Table 1:** Estimated Drug Acquisition Costs of The SHEP Treatment Protocol

Drug Category	Drug Cost Per Patient in 1 Year	Proportion	Drug Cost Per Patient in 5 years
			
step1 dose1 (chlorthalidone 12.5 mg/d)	$10.24	0.43	
step1 dose2 (chlorthalidone 25 mg/d)	$20.48	0.23	
step2 dose1 (chlorthalidone 25 mg/d plus atenolol 25 mg/d or reserpine 0.05 mg/d)	$222.45	0.16	
step2 dose2 (chlorthalidone 25 mg/d plus atenolol 50 mg/d or reserpine 0.1 mg/d)	$221.93	0.17	
			
Weighted SHEP Rx	$83.29	0.91	
KCL	$88.33	0.09	
			
Weighted SHEP Rx including KCL	$91.24		$456
SD	$101.78		
			

Results of the 5 year NNT to prevent one adverse event and its associated cost by event type are shown in Table 2. To prevent one death, the cost for the 5 year NNT was $28,284. In other words, we need to treat 62 patients for 5 years in order to prevent one of them from death and the expected drug acquisition cost for the benefit is $28,284. The cost for the 5 year NNT to prevent one patient from one CVD event of any type is about four times lower than that of death. The cost for the 5 year NNT to prevent one MI is much higher than the cost for preventing one stroke or one CHF. Using the cost to prevent one stroke as the base amount, the incremental cost for the NNT for 5 years to prevent one MI or one CHF was $22,354 and $5,474, respectively.

**Table 2:** NNT and Drug Costs by Adverse Events

Event	Placebo risk	Treatment risk	ARR	NNT	(95% CI)	5-Year NNT	5-year Rx Cost Per Patient	Total Cost	Incremental Cost
									
Death	0.1002	0.0858	0.0144	69	(31 – 319)	62	$456	$28,284	$13,230
CVD	0.1746	0.1147	0.0599	17	(12 – 26)	15	$456	$6,843	-
Stroke	0.0705	0.0433	0.0272	37	(24 – 76)	33	$456	$15,055	$0 (base)
MI	0.0312	0.0202	0.011	91	(48 – 740)	82	$456	$37,408	$22,354
CHF	0.0397	0.0198	0.0199	50	(33 – 103)	45	$456	$20,529	$5,474

Table 3 presents costs of the NNT for 5 years to prevent one CVD event of any type by CVD risk strata. The cost for the 5 year NNT increases as the CVD risk level decreases. It costs $20,529 for the 5 year NNT to prevent one of any type of CVD adverse events among patients in the low CVD risk group. In contrast, it only costs $5,018 for the same effect among patients in the very high CVD risk group. Using the cost of the very high CVD level as a base, if 12 patients in the high CVD level are treated, the extra cost to prevent one patient out of 12 from one CVD event is $456. The extra cost for patients in the low CVD risk group to receive the same effect is $15,511 relative to the patients in the very high CVD risk group.

**Table 3:** NNT and Drug Costs by CVD Risk Profile

Risk Category	Placebo Risk	Treatment Risk	ARR	NNT	(95% CI)	5-year NNT	5-year Drug Cost Per Patient	Total Cost	Incremental Cost
									
1 (low)	0.1013	0.0814	0.0199	50	(18 – 59)	45	$456	$20,529	$15,511
2 (medium)	0.1476	0.0912	0.0564	18	(11 – 53)	16	$456	$7,299	$2,281
3 (high)	0.2044	0.1265	0.0779	13	(8 – 26)	12	$456	$5,474	$456
4 (very high)	0.2526	0.1699	0.0827	12	(7 – 38)	11	$456	$5,018	$0 (base)

In Table 4, the comparisons of the incremental drug acquisition cost for the 5 year NNT of the SHEP-based antihypertensive therapy to other commonly prescribed antihypertensive drugs. This analysis assumes that alternative drugs have equal efficacy to prevent CVD events. The estimated incremental net cost of the 5 year NNT to prevent one CVD event associated with use of atenolol (beta-blocker), enalapril (ACE inhibitor), terazosin (alpha-blocker), and nifedipine (calcium channel blocker) relative to the SHEP-based drug therapy ranged from $6,372 to $38,667 in older adults with isolated systolic hypertension. According to the cost ratio, it indicates that the costs of the 5 year NNT of using enalapril, terazosin, and nifedipine were up to 6.6 times more expensive compared to the SHEP-based drug therapy.

**Table 4:** Comparisons of Drug Acquisition Costs of 5-Year NNT Among Antihypertensive Drug Classes

**Drug Class**	**Commonly Prescribed**	**5-year Cost Per Patient**	**5-Year NNT **	**Total Cost**	**Incremental Cost**	**Cost Ratio **
						
SHEP-based drug therapy		$456	15	$6,843	$0 (base)	1 (base)
						
Beta-Blocker	Atenolol					
	25 mg daily	$1,255	15	$18,825	$11,982	2.75
	50 mg daily	$1,245	15	$18,675	$11,832	2.73
	100 mg daily	$1,792	15	$26,880	$20,037	3.93
						
ACE inhibitor	Enalapril					
	5 mg daily	$2,031	15	$30,465	$23,622	4.45
	10 mg daily	$2,132	15	$31,980	$25,137	4.67
	20 mg daily	$3,034	15	$45,510	$38,667	6.65
						
Alpha-Blocker	Terazosin					
	2 mg daily	$2,984	15	$44,760	$37,917	6.54
	5 mg daily	$2,984	15	$44,760	$37,917	6.54
	10 mg daily	$2,984	15	$44,760	$37,917	6.54
						
Calcium channel blocker	Nifedipine					
	30 mg daily	$881	15	$13,215	$6,372	1.93
	60 mg daily	$1,762	15	$26,430	$19,587	3.86
	90 mg daily	$2,644	15	$39,660	$32,817	5.8

## Discussion

The result of an economic evaluation essentially shows the cost per benefit gained from adapting a specific treatment. The effective and efficient use of resources has been increasingly emphasized from society, health plans, and health care providers. This cost-minimization analysis incorporating outcome data from the SHEP trial presents information treatment cost for older patients with ISH. We found that a long-term, low-dose and diuretic-based antihypertensive therapy is relatively inexpensive and effectively prevents adverse events associated with cardiovascular diseases, especially in older patients who had a high CVD risk profile.

Our findings indicate that the total and incremental treatment costs of antihypertensive drugs in ambulatory care settings range widely among drug classes as well as within drug classes. This analysis suggests that diuretic-based antihypertensive treatments are the least expensive, whereas atenolol (beta-blocker) is less costly than enalapril (ACE inhibitor) and nifedipine (calcium channel blocker), and terazosin (alpha-blocker) is the most expensive drugs in terms of the 5 year NNT to prevent one CVD event. It appears that use of the SHEP-based drug therapy offers greater economic benefits for controlling isolated systolic hypertension in the elderly than other antihypertensive drug treatments. Using a decision analysis model that simulated clinical decisions and outcomes that would occur when primary care physicians follow the JNC IV hypertension management guidelines, it was found that a newer class of calcium channel blockers can slightly increase the proportion of patients who achieve and maintain hypertension control, but at a substantially higher cost than with a generic diuretic drug. [15]

For our analyses, we presumed that all drugs offer equivalent therapeutic benefits. This assumption may have introduced a conservative bias into our primary findings. In fact, randomized controlled trials directly comparing active treatments for hypertension reported that calcium antagonists and doxazosin were inferior to low-dose diuretics or other agents in preventing cardiovascular events, suggesting that the cost-effectiveness of diuretic-based treatments may be even more favorable than estimated in the present study. [15-17] Further, in a meta-analysis of over 27,000 patients, those randomized to calcium antagonists as first-line therapy ran a greater risk of experiencing a myocardial infarction (26% higher risk), congestive heart failure (25% higher risk), and all cardiovascular events combined (10% higher) as compared to those randomized primarily to low-dose diuretics, beta-blockers and ACE inhibitors.[16] Finally, the Antihypertensive and Lipid Lowering treatment to prevent Heart Attack Trial (ALLHAT) recently reported a significantly higher risk of congestive heart failure, stroke, and major cardiovascular events in the doxazosin group than in the chlorthalidone group.[17] It is noteworthy that in this trial, only minimal differences in blood pressure control occurred between treatment groups, suggesting that the magnitude of blood pressure control represents an inadequate marker for comparing the therapeutic benefits of antihypertensive therapies.

With regard to costs projected in our study, it is noteworthy to consider that compared to the SHEP treatments, costs of treatments based on more recently developed antihypertensive agents (than reported here) are likely to be even higher than estimated in the present analyses.

The results of this study are limited to men and women 60 years and older who have isolated systolic hypertension and no presumed contraindication to any one class of antihypertensive medications. One limitation to our study relates to the fact that comparisons were based on costs of monotherapies, while combination therapies are frequently needed to control blood pressure.

The number-needed-to-treat to prevent one adverse outcome has become a widely used measure of treatment benefits in medical community, which is easy for physicians to understand. The shortcomings of NNT are that the outcome measure of an effect is with one dimension- survival probability and that it measures the specified outcome at a single point in time. Therefore, a measure of NNT can not capture an outcome in effectiveness of the intervention with two dimensions: time and survival probability. These limitations may not allow us to take time and discounting on cost and effect into account in this study.

## Conclusion

Based on our findings, antihypertensive therapy that is diuretic-based and that includes either low-dose reserpine or atenolol represents a cost-effective regimen in preventing or delaying cardiovascular events in older adults. Use of the diuretic-based therapy is the most cost-effective in patients at high risk for developing cardiovascular disease. These results suggest that clinicians should consider using diuretics plus low-dose reserpine or atenolol as first-line therapy in patients with isolated systolic hypertension who are greater than 60 years old when there are no contraindications among these patients.

## List of Abbreviations Used

ACE: angiotensin-converting enzyme

ALLHAT: Antihypertensive and Lipid Lowering treatment to prevent Heart Attack Trial

ARR: absolute risk reduction

AWP: average wholesale price

BP: blood pressure

CHD: coronary heart disease

CHF: congestive heart failure

CVD: cardiovascular disease

DBP: diastolic blood pressure

HDL: high density lipid

ISH: isolated systolic hypertension

JNC IV The Sixth Report of the Joint National Committee on Prevention, Detection, Evaluation, and Treatment of High Blood Pressure.

NNT: number-needed to treat

SBP: systolic blood pressure

SHEP: Systolic Hypertension in the Elderly Program

## Conflict of Interest

The author(s) declare that they have no competing interests.

## Authors' contributions

GC, LF, WM and MP participated the development of the analytic framework. GC performed all data analyses. GC, LF, WM and MP drafted and revised the manuscript. All authors approved the final manuscript.
